# Three-dimensional multifrequency magnetic resonance elastography improves preoperative assessment of proliferative hepatocellular carcinoma

**DOI:** 10.1186/s13244-023-01427-4

**Published:** 2023-05-18

**Authors:** Guixue Liu, Di Ma, Huafeng Wang, Jiahao Zhou, Zhehan Shen, Yuchen Yang, Yongjun Chen, Ingolf Sack, Jing Guo, Ruokun Li, Fuhua Yan

**Affiliations:** 1 Department of Radiology, Ruijin Hospital, Shanghai Jiao Tong University School of Medicine, No. 197 Ruijin Er Road, Shanghai, 200025 China; 2 Department of General Surgery, Ruijin Hospital, Shanghai Jiao Tong University School of Medicine, Shanghai, China; 3 Department of Pathology, Ruijin Hospital, Shanghai Jiao Tong University School of Medicine, Shanghai, China; 4 Department of Radiology, Charité–Universitätsmedizin Berlin, Berlin, Germany

**Keywords:** Hepatocellular carcinoma, Proliferative, Magnetic resonance elastography, Magnetic resonance imaging

## Abstract

**Background:**

To investigate the viscoelastic signatures of proliferative hepatocellular carcinoma (HCC) using three-dimensional (3D) magnetic resonance elastography (MRE).

**Methods:**

This prospective study included 121 patients with 124 HCCs as training cohort, and validation cohort included 33 HCCs. They all underwent preoperative conventional magnetic resonance imaging (MRI) and tomoelastography based on 3D multifrequency MRE. Viscoelastic parameters of the tumor and liver were quantified as shear wave speed (*c*, m/s) and loss angle (*φ*, rad), representing stiffness and fluidity, respectively. Five MRI features were evaluated. Multivariate logistic regression analyses were used to determine predictors of proliferative HCC to construct corresponding nomograms.

**Results:**

In training cohort, model 1 (Combining cirrhosis, hepatitis virus, rim APHE, peritumoral enhancement, and tumor margin) yielded an area under the curve (AUC), sensitivity, specificity, accuracy of 0.72, 58.73%,78.69%, 67.74%, respectively. When adding MRE properties (tumor *c* and tumor *φ*), established model 2, the AUC increased to 0.81 (95% CI 0.72–0.87), with sensitivity, specificity, accuracy of 71.43%, 81.97%, 75%, respectively. The C-index of nomogram of model 2 was 0.81, showing good performance for proliferative HCC. Therefore, integrating tumor *c* and tumor *φ* can significantly improve the performance of preoperative diagnosis of proliferative HCC (AUC increased from 0.72 to 0.81, *p* = 0.012). The same finding was observed in the validation cohort, with AUC increasing from 0.62 to 0.77 (*p* = 0.021).

**Conclusions:**

Proliferative HCC exhibits low stiffness and high fluidity. Adding MRE properties (tumor *c* and tumor *φ*) can improve performance of conventional MRI for preoperative diagnosis of proliferative HCC.

**Critical relevance statement:**

We investigated the viscoelastic signatures of proliferative hepatocellular carcinoma (HCC) using three-dimensional (3D) magnetic resonance elastography (MRE), and find that adding MRE properties (tumor* c* and tumor* φ*) can improve performance of conventional MRI for preoperative diagnosis of proliferative HCC.

**Graphical Abstract:**

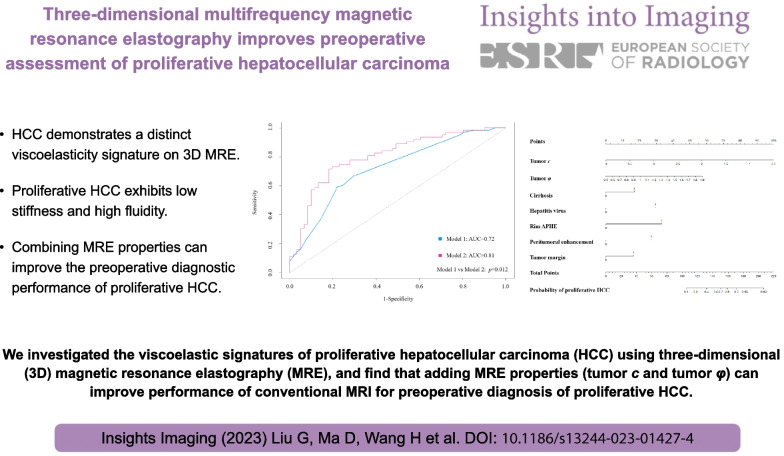

**Supplementary Information:**

The online version contains supplementary material available at 10.1186/s13244-023-01427-4.

## Key points

1. HCC demonstrates a distinct viscoelasticity signature on 3D MRE.
2. Proliferative HCC exhibits low stiffness and high fluidity.
3. Combining MRE properties can improve the preoperative diagnostic performance of proliferative HCC.

**Supplementary Information:**

The online version contains supplementary material available at 10.1186/s13244-023-01427-4.

## Background

Hepatocellular carcinoma (HCC) accounts for 90% of primary liver cancers and is the second most common cause of cancer-related deaths worldwide [1]. HCC exhibits a highly heterogeneous phenotype at the molecular and histologic levels [2, 3]. By integrating morphology and molecular alterations, HCC can be classified as either the proliferative (~ 50%) or non-proliferative (~ 50%) phenotype [2, 4]. Each proliferation class is characterized by activation of varied genomic pathways related to cellular proliferation and survival (e.g., *AKT/mTOR*, MET, TGF-b, and insulin-like growth factor pathways), high rates of chromosomal instability, and aberrant epigenetic changes [2, 5]. Compared with non-proliferative HCC, proliferative HCC demonstrates an invasive phenotype with moderate to poor cellular differentiation, frequent vascular invasion, high tumor recurrence, and a poor prognosis [2, 5]. Therefore, identifying the aggressive HCC subtypes during pretherapeutic work-ups may have strong prognostic and therapeutic implications.

Recent studies have shown that imaging findings can be correlated with specific molecular traits of HCC. Histologically, proliferative HCCs include the progenitor, macrotrabecular, scirrhous, sarcomatoid, and neutrophil-rich types [6]. Progenitor-type HCC usually appears as targetoid dynamic enhancement patterns (LR-M), with more marked hypointensity on the hepatobiliary phase, lower apparent diffusion coefficients, and non-smooth tumor margins [7–9]. A recent study [10] showed that substantial necrosis, high serum AFP levels, and Barcelona Clinic Liver Cancer (BCLC) stage B or C were independent predictors of the macrotrabecular-massive (MTM)-HCC subtype; substantial necrosis helped identify MTM-HCCs with 65% sensitivity. Kang and Kim et al. [11] evaluated the role of gadoxetate-enhanced MRI in differentiating proliferative from non-proliferative HCCs. These authors demonstrated that most proliferative HCCs showed rim APHE (61.9% vs 11.2% for non-proliferative HCCs), with 88.8% specificity. Combining rim APHE and a serum AFP (> 100 ng/mL) for diagnosing proliferative tumors increased the specificity to 98.3% (114/116). However, its sensitivity was 26.2%, which may be insufficient for clinical practice. Additionally, the sample did not include CK7-positive HCC (another significant proliferative type). Collectively, a comprehensive understanding of the imaging features of proliferative HCC remains limited.

Magnetic resonance elastography (MRE) allows noninvasively quantifying tissue mechanical properties in vivo and provides new insights into tumor biology. MRE has been developed to detect and characterize cancers, evaluate therapeutic responses, and investigate the underlying biophysical mechanisms associated with malignant transformation [12, 13]. Tomoelastography based on three-dimensional (3D) MRE techniques use multifrequency data acquisition and a wave-number-based inversion algorithm [14]. It yields highly resolved quantitative maps of shear wave speed (*c*, m/s) and loss angle (*φ*, rad) as surrogates of tissue stiffness and fluidity, respectively [15]. 3D MRE can be used to noninvasively differentiate benign and malignant liver lesions [16] and detect pancreatic and prostate cancers [17, 18]. To our knowledge, the value of 3D MRE for predicting patients with proliferative HCC remains unknown.

Mechanical changes in the liver may be the source of genetic instability leading to tumor development, which can be used to explore the biological mechanism of liver tumor from the perspective of biomechanics [19]. Because proliferative HCC types exhibit underlying histological features, such as abundant fibrous stroma, rich necrotic components, and few tumor pseudocapsules [7–9], we hypothesized that biomechanical properties are sensitive to structural composition and arrangements and may differ between proliferative and non-proliferative HCC. Here, we aimed to identify biomechanical features of proliferative HCCs using 3D MRE properties and developed MRE-based nomogram that can be used in patients with HCC for distinguishing the proliferative and non-proliferative HCC subtypes.

## Materials and methods

### Participants

**Fig. 1:**
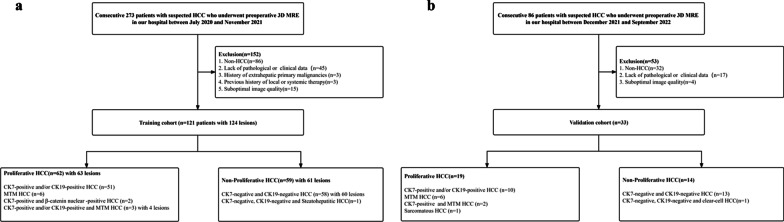
Flowchart of participant inclusions and exclusions. **a** Training cohort. **b** Validation cohort.* HCC* hepatocellular carcinoma;* MTM* macrotrabecular-massive

The institutional review board of our hospital approved this prospective study (No. RJ2018-209), and all participants provided written informed consent. Two hundred seventy-three consecutive patients with focal liver lesions were included from July 2020 to November 2021 and were selected as the training cohort. One hundred fifty-two were excluded for the following reasons: non-HCC on pathology (*n* = 86), lack of pathological results (*n* = 45), suboptimal image quality (*n* = 15), history of extrahepatic primary malignancies (*n* = 3), and history of local or systemic therapy (*n* = 3). Finally, 121 patients with 124 HCC lesions were included (Fig. 1a). Then, 33 HCC patients from December 2021 to September 2022 who met the above criteria were included as validation cohort (Fig. 1b). The time intervals between surgery and MRI examination were within 3 days.

### Conventional MRI

Conventional MRI examinations were performed on machines from three vendors (Magnetom Aera, Siemens, Germany; Ingenia, Philips, Netherlands; UN790, United Imaging, China) equipped with a dedicated 18-channel system. The liver imaging protocol included T1-weighted images (T1WI), T2-weighted images (T2WI), diffusion-weighted imaging (DWI) with b values of 0, 50 and 800 s/mm^2^, and gadolinium-diethylenetriaminepentaacetic acid (Gd-DTPA)-based dynamic contrast-enhanced (DCE) imaging. Additional file 1: Table S1 shows the imaging parameters for the MRI scanning protocol.

### 3D multifrequency MRE

3D multifrequency MRE was performed only on a 1.5-T MRI scanner (Magnetom Aera, Siemens, Erlangen, Germany) where MRE equipments are available. All patients fasted for at least 4 h prior to the examination. Our setup was similar to that described by Shahryari et al. [16]. Briefly, mechanical vibrations of 30, 40, 50 and 60 Hz were generated and transmitted to the liver using four surface-based pressure pads powered by compressed air. Two anterior and two posterior pads centered around the liver region were operated at 0.4 and 0.6 bar, respectively. The 3D wave fields were acquired by single-shot, spin-echo planar MRI sequencing with flow-compensated motion-coding gradients (MEGs).

As per Shahryari et al. [20], 15 contiguous slices with a field of view (FOV) of 312 × 384 mm^2^, matrix size of 104 × 128, and resolution of 3 × 3 × 5 mm^3^ were obtained during free breathing. Further imaging parameters were repetition time (TR) = 2050 ms; echo time (TE) = 59 ms; parallel imaging with GRAPPA factor 2; MEG amplitude = 30 mT/m; and MEG frequencies = 43.48 Hz for 30, 40, and 50 Hz vibration frequencies and 44.88 Hz for 60 Hz vibration frequencies. The total acquisition time was approximately 3.5 min.

Multifrequency wave field data were processed using specialized software available at https://bioqic-apps.com. Finally, the FOV maps including shear wave speed (*c*) and loss angle (*φ*) of the complex shear modulus were generated. As *c* is directly proportional to the square root of the storage modulus (the real part of the complex shear modulus), and *φ* continuously changes from 0 (pure solid properties) to *π*/2 (pure fluid property), these two parameters were considered substitutes for stiffness and tissue fluidity, respectively. Herein, we use *c* and *φ* to describe quantitative information, and “stiffness” and “fluidity” to describe changes in qualitative parameters.

### Image analysis

Two experienced radiologists (reader 1 with 15 years of experience and reader 2 with 3 years of experience) reviewed all preoperative MRI features in consensus. For each lesion, the readers independently evaluated the following imaging features of each HCC: (a) rim arterial phase hyperenhancement (rim-APHE), defined as rim-like enhancement with relatively hypovascular central areas in the arterial phase [11, 22]; (b) nonperipheral washout, defined as the reduction of overall or partial enhancement of nonperipheral visual assessment relative to composite liver parenchyma according to LI-RADS version 2018 [11, 21]; (c) capsule, defined as linear, thin and enhanced peripheral rim of smooth hyper-enhancement in the portal venous or delayed phase, according to LI-RADS version 2018 [21–23]; (d) peritumoral enhancement, defined as polygonal or crescent shaped enhancement that can be detected outside the tumor margin, which has a broad contact with the tumor boundary in the arterial phase, and shows same intensity with the background liver parenchyma in the equilibrium or portal venous phase [22, 23]; and (e) tumor margins which are defined at the equilibrium phase or portal venous phase can be divided into: smooth margin, showing as a nodular tumor with smooth contour; non-smooth tumor margin, presenting as non-smooth nodular tumors with focal extranodular growth [11, 22, 23]. If no consensus could be reached, a third reader (reader 3 with 32 years of experience) was consulted for the final decision.

For viscoelasticity measurements, the region of interest (ROI) was drawn manually based on magnitude images. A main slice showing the primary lesion at the maximum cross-sectional extension and two adjacent slices were selected to determine the ROIs. ROIs were defined to include only the tumor and liver parenchyma while avoiding the boundaries, tissue interface and large blood vessels. The readers were blinded to all clinical and laboratory information and histopathological results.

### Histopathological analysis

A pathologist with 15 years of experience who was blinded to all radiological and clinical results analyzed all specimens. Histomorphological subtypes were classified according to the World Health Organization (2019) [24]. Immunohistochemical staining for CK19 and CK7 was performed on representative tissue sections. When > 5% of tumor cells expressed CK19, the HCC was determined to be CK19-positive [25]. When > 5% of tumor cells were immunoreactive, they were considered CK7-positive [26]. When > 50% of the tumor showed a major trabecular structural pattern, it was defined as MTM [5]. CK19-positive and CK7-positive, MTM, neutrophil-rich, sclerosing, and sarcomatous HCCs were classified as proliferative HCCs; CK19-negative and CK7-negative, steatohepatitic, lymphocyte-rich, and clear-cell HCCs were classified as non-proliferative HCCs [2].

### Statistical analysis

For intergroup comparisons, continuous variables were tested using Student’s t-test or the Mann–Whitney U test based on normality. Categorical variables were tested via the chi-square test or Fisher’s exact test. Intraclass correlation coefficients (ICCs) were used to test measurement consistency between two observers. Multivariate analysis with backward logistic regression was used to identify variables that were significantly and independently associated with proliferative HCC (dependent variable), and corresponding prediction nomograms were constructed. Variables included in the multivariate analysis were those significantly associated with proliferative HCC in the univariate analysis, as well as those thought to impact clinical outcomes, and included age, BMI, size, and MRE properties (tumor *c* and tumor *φ*) as continuous variables and sex, AFP, cirrhosis, hepatitis virus, major imaging features (rim APHE, nonperipheral washout, capsule, peritumoral enhancement, tumor margin), as dichotomous variables. Receiver operating characteristic curve analysis was performed to determine the diagnostic performance for predicting proliferative HCC. Decision curve analysis (DCA) was used to evaluate the clinical net benefits of the nomogram. All statistical analyses were performed using SPSS (version 26 for Windows; SPSS, Chicago IL, USA) and R (v4.0.5; URL http://www.r-project.org); *p* < 0.05 was considered statistically significant.

## Results

### Demographic characteristics

In the training cohort, of 121 patients with 124 HCC lesions, 62 had proliferative HCC (mean age, 58 ± 12 years; 51 men, 11 women, 63 lesions), and 59 had non-proliferative HCC (mean age, 61 ± 10 years; 50 men, 9 women; 61 lesions). Cirrhosis differed significantly between proliferative and non-proliferative HCCs (*p* = 0.03). The proliferative HCCs were significantly smaller than the non-proliferative HCCs (3.69 ± 3.22 vs. 4.83 ± 3.63 cm, *p* = 0.01). On histopathology, the proliferative HCCs were classified as CK7-positive (19.35%, 24/124), CK19-positive (10.48%, 13/124), CK7-positive and CK19-positive (11.29%, 14/124), MTM subtype (4.84%, 6/124), CK7-positive and β-catenin nuclear-positive (1.61%, 2/124), or CK7-positive and/or CK19-positive and MTM (3.23%, 4/124). The remaining HCCs were non-proliferative and included CK7-negative and CK19-negative HCCs (48.39%, 60/124), and CK7-negative, CK19-negative and steatohepatitic HCCs (0.81%, 1 of 124). In the validation cohort, of 33 patients, 19 had proliferative HCCs (mean age, 57 ± 11 years; 17 men, 2 women), and 14 had non-proliferative HCCs (mean age, 55 ± 11 years; 12 men, 2 women). Size differed significantly between proliferative and non-proliferative HCCs (*p* = 0.021). On histopathology, the proliferative HCCs were classified as CK7-positive (24.24%, 8/33), CK7-positive and CK19-positive (6.06%, 2/33), MTM subtype (18.18%, 6/33), CK7-positive and MTM (6.06%, 2/33), sarcomatous HCCs (3.03%, 1/33). The remaining HCCs were non-proliferative and included CK7-negative and CK19-negative HCCs (39.39%, 13/33), and CK7-negative, CK19-negative and clear-cell HCCs (3.03%, 1 of 33). Table 1 describes the patients’ clinicopathological characteristics. There were no significant differences in clinical characteristics between the training and validation cohorts (Additional file 1: Table S2).

**Table 1:** Comparison of the clinicopathological characteristics of proliferative and non-proliferative HCCs

Characteristic	Training cohort (*n* = 121)		*p* value	Validation cohort (*n* = 33)		*p* value
	Proliferative HCC (*n* = 62)	Non-proliferative HCC (*n* = 59)		Proliferative HCC (*n* = 19)	Non-proliferative HCC (*n* = 14)	
*Demographic*						
Age (years) (mean ± SD)	58 ± 12	61 ± 10	0.45	57 ± 11	55 ± 11	0.57
Sex (male: female)	51:11	50:9	0.71	17:2	12:2	0.74
BMI (kg/m^2^)	24.06 ± 3.2	23.85 ± 3.36	0.72	24.09 ± 2.88	24.96 ± 3.58	0.45
Cirrhosis	36 (58.06%)	46 (77.97%)	0.03*	14 (73.68%)	9 (64.29%)	0.56
Etiology			0.24			0.75
Hepatitis virus	56 (90.43%)	49 (83.05%)		14 (73.68%)	11 (78.57%)	
Non-hepatitis virus	6 (9.68%)	10 (16.95%)		5 (26.32%)	3 (21.43%)	
*Preoperative laboratory results*						
Albumin (g/dL)	39.77 ± 4.44	38.75 ± 6.12	0.08	37.84 ± 5.45	40.21 ± 2.42	0.14
Total bilirubin (µmol/L)	20.04 ± 14.96	18.87 ± 14.69	0.49	24.42 ± 15.03	17.31 ± 9.01	0.13
AFP (ng/mL)			0.99			0.89
≤ 100	42 (67.74%)	40 (67.80%)		14 (73.68%)	10(71.43%)	
> 100	20 (32.26%)	19 (32.20%)		5 (26.32%)	4 (28.57%)	
CEA (ng/mL)			0.96			0.21
> 5	2 (3.23%)	2 (3.39%)		2 (10.53%)	0	
≤ 5	60 (96.77%)	57 (96.61%)		17 (89.47%)	14 (100%)	
CA125 (U/mL)			0.39			0.07
> 24	12 (19.35%)	8 (13.56%)		4 (21.05%)	0	
≤ 24	50 (80.65%)	51 (86.44%)		15 (78.95%)	14 (100%)	
CA199 (U/mL)			0.71			0.75
> 25	17 (27.42%)	18 (30.51%)		5 (26.32%)	3 (21.43%)	
≤ 25	45 (72.58%)	41 (69.49%)		14 (73.68%)	11 (78.57%)	
INR unit	1.055 ± 0.11	1.03 ± 0.11	0.12	1.08 ± 0.13	1.06 ± 0.07	0.87
ALT (IU/L)	42.52 ± 38.23	35.10 ± 43.79	0.28	54.37 ± 63.91	28.5 ± 13.37	0.22
AST (IU/L)	45.50 ± 42.36	44.81 ± 46.56	0.75	53.63 ± 63.17	28.57 ± 8.26	0.33
*HCC lesion features*						
Number of lesions	63	61		19	14	
Size (cm)	3.69 ± 3.22	4.83 ± 3.63	0.01*	4.55 ± 2.75	2.95 ± 2.98	0.021*

Data are the mean ± standard deviation or median and interquartile range (IQR) unless otherwise indicated

*HCC* hepatocellular carcinoma; *BMI* body mass index; *AFP α*-fetoprotein; *CEA* carcinoembryonic antigen; *CA125* carbohydrate antigen 125; *CA199* Carbohydrate antigen 199; *INR* international normalized ratio of prothrombin time; *ALT* Alanine transaminase; *AST* Aspartate transaminase

*Indicates statistically significant *p* values

### Major features and LI-RADS categories

**Fig. 2:**
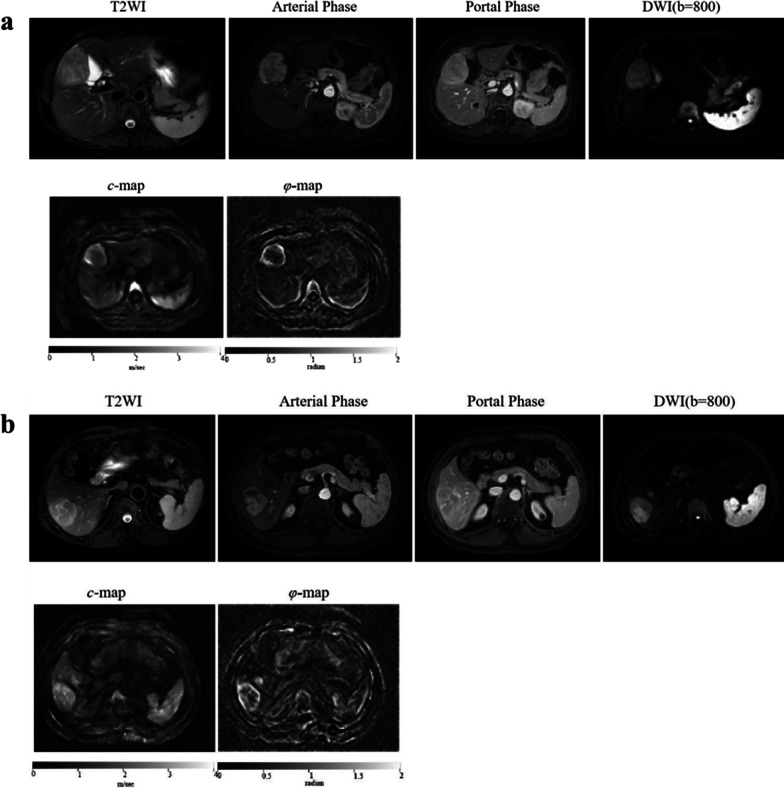
Representative patients’ images. Axial T2-weighted images, arterial-phase images, portal-phase images, diffusion-weighted images at a b value of 800 s/mm^2^, axial *c* and *φ* maps of tumors obtained in (**a**) a patient with proliferative HCC (tumor *c*: 2.31 m/s, tumor *φ*: 1.23 rad, liver *c*: 1.91 m/s, and liver *φ*: 0.68 rad) and (**b**) a patient with non-proliferative HCC (tumor *c*: 2.51 m/s, tumor *φ*: 1.11 rad, liver *c*: 1.65 m/s, and liver *φ*: 0.79 rad)

In the training cohort, rim APHE (*p* = 0.02) and LI-RADS categorization (*p* = 0.005) differed significantly between proliferative and non-proliferative HCC. No significant differences were found for nonperipheral washout (*p* = 0.89), capsule (*p* = 0.24), peritumoral enhancement (*p* = 0.27), or tumor margin (*p* = 0.09). Figure 2 shows representative images of proliferative and non-proliferative HCCs. Table 2 lists the image features and LI-RADS categories.

**Table 2:** Major imaging features and LI-RADS categories for proliferative and non-proliferative HCC

	Proliferative HCC (*n* = 63)	Non-proliferative HCC (*n* = 61)	*p* value
Rim APHE			0.02*
Absent	51 (80.95%)	58 (95.08%)	
Present	12 (19.05%)	3 (4.92%)	
Nonperipheral washout			0.89
Absent	24 (38.10%)	24 (39.34%)	
Present	39 (61.90%)	37 (60.66%)	
Capsule			0.24
Absent	26 (41.27%)	19 (31.15%)	
Present	37 (58.73%)	42 (68.85%)	
Peritumoral enhancement			0.27
Absent	56 (88.89%)	50 (81.97%)	
Present	7 (11.11%)	11 (18.03%)	
Tumor margin			0.09
Smooth	33 (52.38%)	41 (67.21%)	
Non-smooth	30 (47.62%)	20 (32.79%)	
LR-RADS categorization			0.005*
LR-M	12 (19.05%)	3 (4.92%)	
LR-TIV	4 (6.35%)	1 (1.64%)	
LR-3	7 (11.11%)	2 (3.28%)	
LR-4	13 (20.63%)	10 (16.39%)	
LR-5	27 (42.86%)	45 (73.77%)	

*APHE* arterial-phase hyperenhancement; *LI-RADS* Liver Imaging Reporting & Data System; *TIV* tumor in vein

*Indicates statistically significant *p* values

### Viscoelasticity signatures of proliferative HCC

The ICCs were 0.96 (95% confidence interval [95% CI] 0.94–0.97) for tumor *c*, 0.88 (95% CI 0.84–0.92) for tumor *φ*, 0.95 (95% CI 0.93–0.97) for liver *c*, and 0.89 (95% CI 0.84–0.92) for liver *φ*, indicating good reproducibility (Additional file 1: Figure S1).

In the training cohort, Table 3 lists the biomechanical properties of proliferative and non-proliferative HCC. Tumor *c* was lower for proliferative HCC than for non-proliferative HCC (2.13 ± 0.58 vs. 2.36 ± 0.60 m/s, *p* = 0.03), indicating that proliferative HCCs were softer than were non-proliferative HCCs. However, tumor *φ*, liver *c*, and liver *φ* did not significantly differ between the two groups. 80.9% (51/63) of proliferative HCCs were progenitor-type (CK7- and/or CK19-positive). Further subgroup analysis between progenitor-type and non-progenitor-type HCCs showed similar results (Additional file 1: Table S3). Tumor *c* was lower for progenitor-type HCC than for non-progenitor-type HCC (2.10 ± 0.60 vs. 2.36 ± 0.61 m/s, *p* = 0.02). Tumor *φ*, liver *c*, and liver *φ* did not significantly differ between the subgroups.

**Table 3:** Mechanical parameters *c* (stiffness) and *φ* (fluidity) of proliferative and non-proliferative HCC

Parameters	Proliferative HCC (*n* = 63)	Non-proliferative HCC (*n* = 61)	*p* value
*Tumor*			
*c* (m/s)	2.13 ± 0.58	2.36 ± 0.60	0.03*
*φ* (rad)	1.08 ± 0.22	1.06 ± 0.21	0.64
*Liver*			
*c* (m/s)	1.94 ± 0.41	2.05 ± 0.42	0.18
*φ* (rad)	0.79 ± 0.12	0.77 ± 0.15	0.26

*Indicates statistically significant *p* values

### MRE-based prediction model for proliferative HCC

**Fig. 3:**
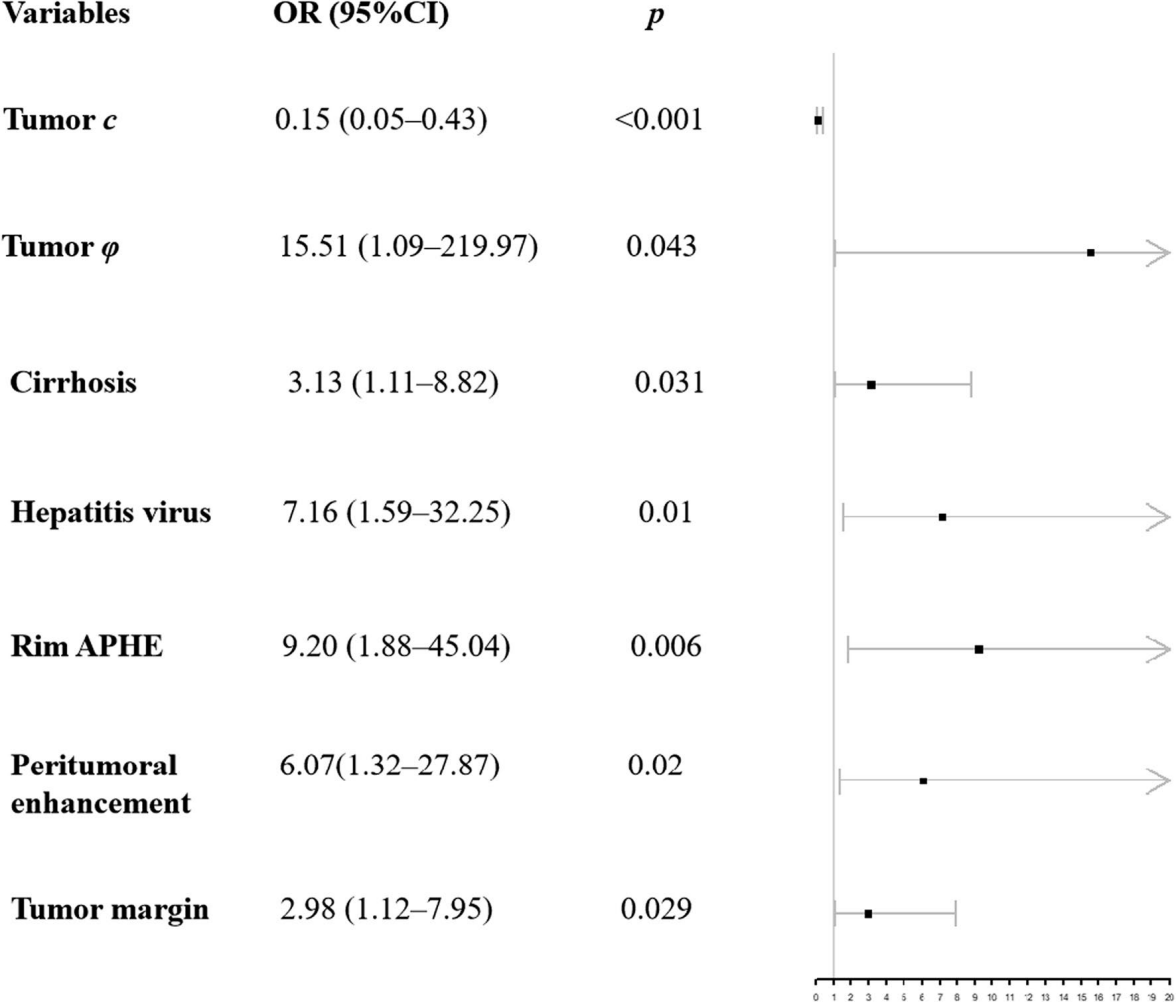
Forest plot of independent predictors of proliferative HCC with a multivariate regression model. Lines represent the 95%CI for tumor *c*, tumor *φ*, cirrhosis, hepatitis virus, rim APHE, peritumoral enhancement, and tumor margin. Squares represent the OR for tumor *c*, tumor *φ*, cirrhosis, hepatitis virus, rim APHE, peritumoral enhancement, and tumor margin. APHE: arterial phase hyperenhancement; OR: odds ratio; CI: confidence interval

In the training cohort, univariate logistic regression analysis showed that tumor *c* (odds ratio [OR]: 0.51, 95% CI 0.27–0.95; *p* = 0.035), cirrhosis (OR: 2.3, 95% CI 1.07–4.95; *p* = 0.033), and rim APHE (OR: 4.55, 95% CI 1.22–17.03; *p* = 0.024) were associated with proliferative HCC (Table 4). To adjust for confounding variables, multivariate analysis with backward stepwise regression showed that tumor *c* (OR: 0.15, 95% CI 0.05–0.43; *p* < 0.001), tumor *φ* (OR: 15.51, 95% CI 1.09–219.97; *p* = 0.043), cirrhosis (OR: 3.13, 95% CI 1.11–8.82; *p* = 0.031), hepatitis virus (OR: 7.16, 95% CI 1.59–32.25; *p* = 0.01), rim APHE (OR: 9.20, 95% CI 1.88–45.04; *p* = 0.006), peritumoral enhancement (OR: 6.07, 95% CI 1.32–27.87; *p* = 0.02), and tumor margin (OR: 2.98, 95% CI 1.12–7.95; *p* = 0.029) were independent factors for proliferative HCC (Fig. 3).

**Table 4:** Univariate and multivariate analysis of variables associated with proliferative HCC

	Univariate		Multivariate	
	OR (95% CI)	*p* value	OR (95% CI)	*p* value
Tumor *c* (m/s)	0.51 (0.27–0.95)	0.035*	0.15 (0.05–0.43)	< 0.001*
Tumor *φ* (rad)	1.64 (0.31–8.63)	0.56	15.51 (1.09–219.97)	0.043*
*Sex*				
Female	1 (Reference)			
Male	0.93 (0.36–2.37)	0.87		
Age	0.98 (0.95–1.01)	0.27		
BMI	1 (0.9–1.11)	1.00		
Size	0.91 (0.82–1.02)	0.07		
*AFP (ng/mL)*				
≤ 100	1 (Reference)			
> 100	1.11 (0.52–2.35)	0.80		
*Cirrhosis*				
Absent	1 (Reference)			
Present	2.3 (1.07–4.95)	0.033*	3.13 (1.11–8.82)	0.031*
*Hepatitis virus*				
Absent	1 (Reference)			
Present	1.86 (0.63–5.49)	0.26	7.16 (1.59–32.25)	0.01*
*Rim APHE*				
Absent	1 (Reference)			
Present	4.55 (1.22–17.03)	0.024*	9.20 (1.88–45.04)	0.006*
*Nonperipheral Washout*				
Absent	1 (Reference)			
Present	1.05 (0.51–2.17)	0.89		
*Capsule*				
Absent	1 (Reference)			
Present	0.64 (0.31–1.35)	0.24		
*Peritumoral enhancement*				
Absent	1 (Reference)			
Present	1.76 (0.63–4.89)	0.28	6.074 (1.32–27.87)	0.02*
*Tumor margin*				
Smooth	1 (Reference)			
Non-smooth	1.86 (0.9–3.86)	0.094	2.98 (1.12–7.95)	0.029*

*OR* odds ratio; *CI* confidence interval; *BMI* body mass index; *A FPα*-fetoprotein; *APHE* arterial phase hyperenhancement

*Indicates statistically significant *p* values

**Fig. 4:**
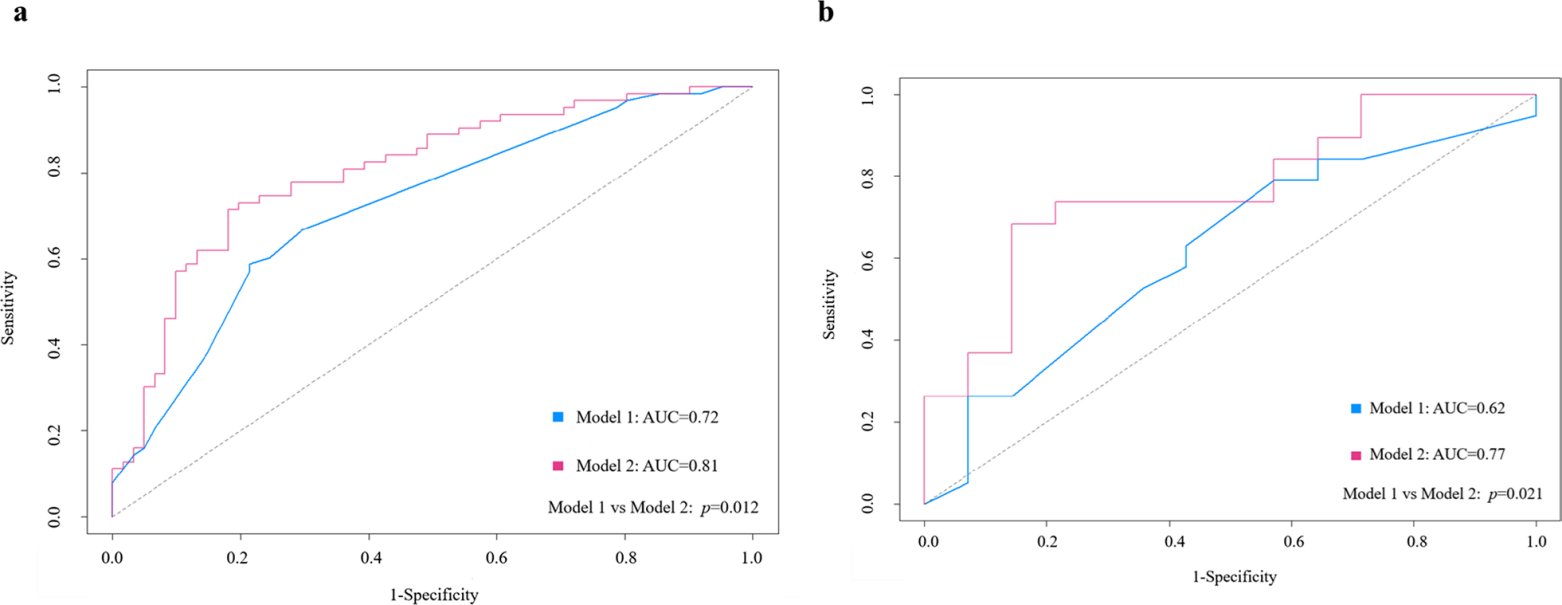
Area under the receiver operating characteristic curve for predicting proliferative HCC. **a** Model 1 (Combining cirrhosis, hepatitis virus, rim APHE, peritumoral enhancement, and tumor margin) and model 2 (Combining tumor *c*, tumor *φ*, cirrhosis, hepatitis virus, rim APHE, peritumoral enhancement, and tumor margin) in the training cohort. **b** Model 1 and model 2 in the validation cohort. APHE: arterial phase hyperenhancement

**Fig. 5:**
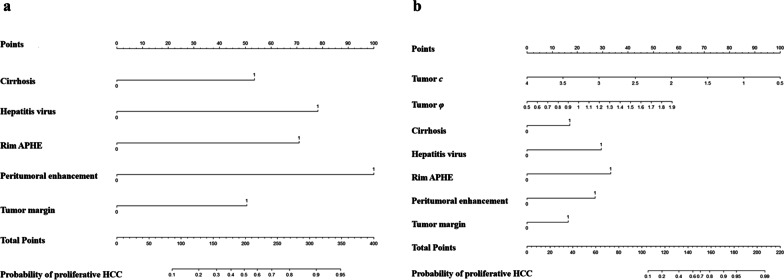
The nomograms yielded for prediction of proliferative HCCs. The nomograms of model 1 (**a**) (Combining cirrhosis, hepatitis virus, rim APHE, peritumoral enhancement, and tumor margin), and model 2 (**b**) (Combining tumor *c*, tumor *φ*, cirrhosis, hepatitis virus, rim APHE, peritumoral enhancement, and tumor margin) for predicting proliferative HCCs*.* For cirrhosis, hepatitis virus, rim APHE, peritumoral enhancement, and tumor margin, “1” refers to presence. “Total Points” is the total score from adding all single scores obtained by variables of models, respectively. Single scores were obtained by drawing a line straight up from a single feature axis to the point axis. At the bottom of the scale, the points of all variables were added to obtain the prediction probability of proliferative HCCs. APHE: arterial phase hyperenhancement

**Fig. 6:**
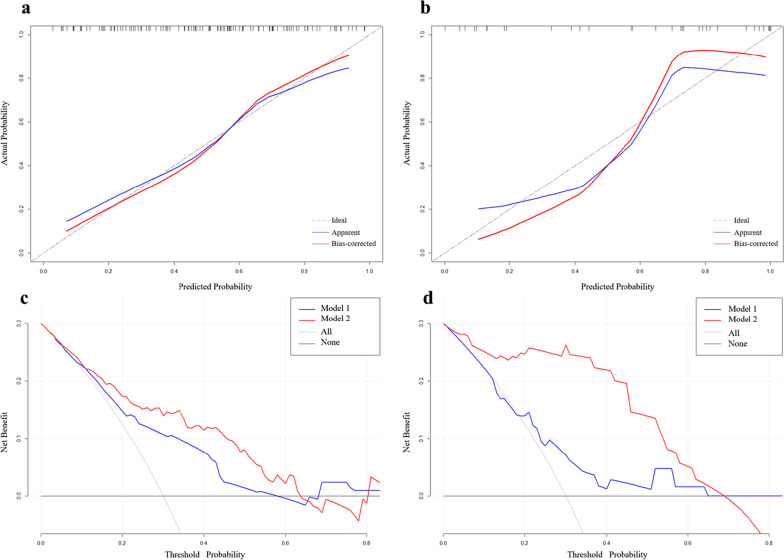
Calibration curves and decision curve analysis of the nomograms in the training and validation cohorts. Calibration curves for the estimation of model 2 (Combining tumor *c*, tumor *φ*, cirrhosis, hepatitis virus, rim APHE, peritumoral enhancement, and tumor margin) predicted by the nomogram in the training cohort (**a**) and in the validation cohort (**b**). The x-axis indicates the predicted probability. The y-axis measures the actual probability. The blue line represents the performance of the nomogram, whereas the red line corrects for any bias in the nomogram. The dashed line represents the reference line where an ideal nomogram would lie. Decision curve analysis (DCA) of model 1 (Combining cirrhosis, hepatitis virus, rim APHE, peritumoral enhancement, and tumor margin) and model 2 in the training cohort (**c**) and in the validation cohort (**d**) indicated their clinical net benefits. The x-axis represents the threshold probability. The y-axis indicates the net benefit. The gray line shows the net benefit of proliferative HCCs. The blue and red lines represented model 1 and model 2 of nomograms, respectively. DCA indicated their clinical net benefits. DCA showed that the model 2 gained more clinical net benefit than the model 1 in both training and validation cohorts. APHE: arterial phase hyperenhancement

In training cohort, model 1 (Established using cirrhosis, hepatitis virus, rim APHE, peritumoral enhancement, and tumor margin) yielded an area under the curve (AUC), sensitivity, specificity, and accuracy of 0.72 (95% CI 0.64–0.80), 58.73 (95% CI 45.6–71.0), 78.69 (95% CI 66.3–88.1), and 68.55 (95% CI 58.76–75.85), respectively (Fig. 4a). The C-index of the regression coefficient-based nomogram of model 1 was 0.72 (95% CI 0.64–0.80), showing good performance for predicting proliferative HCC (Fig. 5a). When adding MRE properties (tumor *c* and tumor *φ*), established model 2, the AUC increased to 0.81 (95% CI 0.72–0.87), with sensitivity, specificity, and accuracy of 71.43 (95% CI 58.7–82.1), 81.97 (95% CI 70.0–90.6), and 76.61 (95% CI 66.43–82.34), respectively (Fig. 4a). The C-index of the regression coefficient-based nomogram of model 2 was 0.81 (95% CI 0.72–0.87), showing good performance for predicting proliferative HCC (Fig. 5b). Therefore, integrating tumor *c* and tumor *φ* can significantly improve the performance for preoperative diagnosis of proliferative HCC (AUC increased from 0.72 to 0.81, *p* = 0.012, Fig. 4a). In the validation cohort, model 1 yielded AUC, sensitivity, specificity, and accuracy of 0.62 (95% CI 0.52–0.68), 63.16 (95% CI 50.03–75.43), 57.14 (95% CI 44.75–66.55), and 60.61 (95% CI 50.82–67.91), respectively (Fig. 4b). When adding MRE properties (tumor *c* and tumor *φ*), established model 2, the AUC increased to 0.77 (95% CI 0.6–0.93), with sensitivity, specificity, and accuracy of 57.89 (95% CI 44.98–68.56), 85.71 (95% CI 73.74–94.34), and 69.70 (95% CI 59.52–75.43), respectively (Fig. 4b). So, integrating tumor *c* and tumor *φ* can significantly improve the performance for preoperative diagnosis of proliferative HCC (AUC increased from 0.62 to 0.77, *p* = 0.021, Fig. 4b). To sum up, in both training and validation cohort, the AUC of model 2 was larger than that of model 1 (all *p* < 0.05) (Fig. 4). The calibration curves of the nomograms showed good consistencies between the predicted and actual probability of proliferative HCCs in both training and validation cohorts of model 2 (Figs. 6a, b). In summary, the nomogram for predicting proliferative HCC in patients with HCC had considerable discriminative and calibrating abilities. In addition, decision curve analysis (DCA) was used to compare and visualize the clinical net benefits of the models (Fig. 6c, d) and showed that the model 2 gained more clinical net benefits than the model 1 in both training and validation cohorts.

## Discussion

In this study, we investigated the viscoelastic signatures of proliferative HCC by 3D MRE. Our results showed that proliferative HCC had decreased tumor *c* and increased tumor *φ* compared with those of non-proliferative HCC. MRE properties combined with conventional imaging and clinical features can significantly improve the performance for preoperative diagnosis of proliferative HCCs.

The softer mechanical signature of proliferative HCC in our study might be associated with its high metastatic potential and invasiveness. Studies have reported that during cancer progression, invasive and metastatic tumor cells may adaptively soften to facilitate and promote migration through narrow tissue space [27, 28]. Epithelial mesenchymal transformation was also reported to soften cancer cells to infiltrate the matrix environment [29, 30]. Rianna et al. found that in 3D cultures, cells softened significantly during channel crossing, and the intracellular stiffness was negatively correlated with invasiveness [27]. The aforementioned mechanical alterations at the cellular level could be manifested collectively as macroscopic tumor softening, as shown here for proliferative HCCs. As reported [16], compared to benign lesions, malignant tumors behaved more fluid-like, showing stronger wave-attenuating properties as a result of changes in vasculature and extracellular matrix network organization, or the presence of necrotic tissue. Therefore, even if the univariate logistic regression analysis yielded no significant correlation, we included tumor *φ* in the multivariate logistic regression analysis. In the multivariate analysis, tumor *φ* was an independent predictor of proliferative HCC. That is, proliferative HCC behaved more fluidly than did non-proliferative HCC. Solid tumors become invasive through metastatic diffusion, which requires partial fluidization for cancer cells to migrate [16]. The increased fluidity may be related to angiogenesis, altered vascular density and increased mechanical friction [16].

Our results showed that cirrhosis is an independent risk factor associated with proliferative HCC. Increased matrix rigidity can promote cancer cell survival, proliferation and migration [31]. Cirrhosis may promote HCC development by paving a collagen-rich rigid “highway” for cancer cell migration [32, 33]. Cirrhotic livers are reported to form immune-mediated cancer fields that contribute to HCC development, as shown by numerous gene signatures obtained from cirrhotic liver tissues [1, 34].

Consistent with previous reports, our results showed that rim APHE is another independent risk predictor of proliferative HCC. Kang and Kim et al. [11] reported that rim APHE was an independent predictor of proliferative HCC and more common in HCC with MTM, CK19-positive, scirrhous and sarcomatoid subtypes [10]. Rhee et al. [35] showed that HCC with rim APHE expressed higher levels of carbonic anhydrase IX and epithelial cell adhesion molecules, which were markers related to hypoxia and stemness, respectively. Rim APHE is related to poor differentiation, frequent microvascular invasion and a poor prognosis [36]. Additionally, most proliferative HCCs in our cohort were CK7-positive and/or CK19-positive. CK7 and CK19 are important markers of liver progenitor cells. Thus, CK7-positive and CK19-positive HCC may have an intermediate phenotype between mature hepatocyte differentiation and the biliary tract during multistep hepatocarcinogenesis [25]. A previous study [35] confirmed that irregular rim-like enhanced HCC may express higher CK19 levels and demonstrate more invasive features.

Non-smooth tumor margins and peritumoral enhancement may reflect the aggressiveness of the HCC [22, 37, 38]. Therefore, even in the univariate logistic regression analysis, tumor margin and peritumoral enhancement were not significantly correlated with proliferative HCC, and we included these two image features in the multivariate logistic regression analysis. The results showed that the non-smooth tumor margin and peritumoral enhancement were independent predictors of proliferative HCC. The non-smooth tumor margin on MRI is a feature of CK19-positive HCC [8, 35], which may be due to the tendency of HCC with a progenitor cell phenotype to have more aggressive growth types and higher histological grades, which may eventually lead to the appearance of non-smooth tumor margins [7]. Studies [39, 40] have shown that peritumoral enhancement is an independent predictor of higher pathological grades. Peritumoral enhancement is considered a useful imaging predictor for early recurrence of HCC after a hepatectomy [38]. Peritumoral enhancement is an important indicator to predict microvascular invasion (MVI) [22, 41–43], which may reflect changes in hemodynamic perfusion during compensatory arterial hyperperfusion [43].

Our study had several limitations. First, this was a single-center exploratory study. Further multicenter validation is needed. Second, our nomogram was established on patients with histologically proven HCC in the dominant areas of viral hepatitis, which limited the application of our nomogram to HCC cohorts only. To expand the clinical utility of our model, our nomogram will be updated in the future by including more HCCs with different etiologies as well as non-HCC lesions. Third, owing to the limited availability of MRE equipment, the clinical MRI examinations were performed on machines from three vendors. However, LI-RADS categories are based on qualitative interpretation of MRI and are system-independent. Finally, proliferative HCCs and non-proliferative HCCs were classified based on histopathological analyses. Further study based on genomic profiling is needed.

## Conclusion

In conclusion, proliferative HCC exhibits a distinct biomechanical signature of low stiffness and high fluidity compared with that of non-proliferative HCC. MRE properties combined with conventional imaging and clinical features can significantly improve the performance of conventional MRI for preoperative diagnosis of proliferative HCCs.

## Supplementary Information

**Additional file 1**. **Supplementary Table 1.** Multiparametric MRI protocol. **Supplementary Table 2.** Demographics and clinical characteristics of the participants in this study. **Supplementary Table 3.** Mechanical parameters c (stiffness) and φ (fluidity) of progenitor-type and non-progenitor-type HCC. **Supplementary Figure 1.** Bland-Altman plots of reader agreement. The c (a) and φ (b) values of the tumor and c (c) and φ (d) values of the liver.

## Data Availability

The datasets used for analyses during the current study are available from the corresponding author on reasonable request.

## Abbreviations

AFP: *α*-Fetoprotein
APHE: Arterial phase hyperenhancement
AUC: Area under the curve
HCC: Hepatocellular carcinoma
ICC: Intraclass correlation coefficient
MRE: Magnetic resonance elastography
MRI: Magnetic resonance imaging
MTM: Macrotrabecular-massive
OR: Odds ratio
ROI: Region of interest
