# Supplementary material for: Three-dimensional multifrequency magnetic resonance elastography improves preoperative assessment of proliferative hepatocellular carcinoma
Source: Insights Imaging. 2023 May 18;14:89. doi: 10.1186/s13244-023-01427-4 (PMC10192481; doi:10.1186/s13244-023-01427-4)

Three-dimensional multifrequency magnetic resonance elastography improves preoperative assessment of proliferative hepatocellular carcinoma

Supplementary Table 1. Multiparametric MRI protocol.

	**Philips**					
	T1W	*T2W*		*DWI*	*DCE*	
	Axial	*Axial*	*Coronal*	*Axial*	*Axial*	*Coronal*
TR (msec)	3.6	2000	1100	1520	3.7	5.2
TE (msec)	1.31	66	80	70	1.32	1.07
Flip angle (degrees)	10	90	90	90	10	10
Field of view (mm)	400×350	360×360	450×450	327×399	400×352	380×380
Matrix	252×217	288×288	320×282	108×128	268×234	212×212
Section thickness (mm)	4	5	5	5	4	4.4
	**Siemens**					
	T1W	*T2W*		*DWI*	*DCE*	
	Axial	*Axial*	*Coronal*	*Axial*	*Axial*	*Coronal*
TR (msec)	4.85	3000	1200	6600	4.85	3.90
TE (msec)	2.41	95	91	62	2.41	1.49
Flip angle (degrees)	10	140	180	180	10	10
Field of view (mm)	380×309	380×380	380×380	380×306	380×3093	360×360
Matrix	320×240	320×320	256×256	134×134	20×240	320×234
Section thickness (mm)	3	5	6	5	3	3
	**United Imaging**					
	T1W	*T2W*		*DWI*	*DCE*	
	Axial	*Axial*	*Coronal*	*Axial*	*Axial*	*Coronal*
TR (msec)	120	4000	1200	4258	4.15	4.31
TE (msec)	2.28	79.92	106.04	75	1.86	1.96
Flip angle (degrees)	70	90	125	90	10	10
Field of view (mm)	410×280	380×360	400×380	380×300	400×300	360×350
Matrix	288×80	320×90	256×85	128×100	256×80	256×73
Section thickness (mm)	6	6	7	6	5	6

Note: b values for DWI were 0, 50, and 800 s/mm^2. T1WI: T1-weighted images; T2WI: T2-weighted images; DWI: diffusion-weighted imaging; DCE: dynamic contrast-enhanced; TE: echo time; TR: repetition time.

Supplementary Table 2. Demographics and clinical characteristics of the participants in this study.

Characteristic	Training cohort (n=121)	Validation cohort (n=33)	*p* value
**Demographic**			
Age (years) (mean ± SD)	59 ± 11	56±11	0.07
Sex (male: female)	101:20	29:4	0.54
BMI (kg/m^2)	23.96 ± 3.27	24.46±3.18	0.43
Cirrhosis	81(66.94%)	23 (69.7%)	0.77
Etiology			0.12
Hepatitis virus	105 (86.78%)	25(75.76%)	
Non-hepatitis virus	16 (13.22%)	824.24%)	
**Preoperative laboratory results**			
Albumin (g/dL)	39.27 ± 5.33	38.85 ± 5.53	0.68
Total bilirubin (µmol/L)	19.47 ± 14.78	21.4 ± 13.15	0.54
AFP (ng/mL)			0.59
≤100	82 (67.77%)	24 (72.73%)	
>100	39 (32.23%)	9 (27.27%)	
CEA (ng/mL)			0.47
>5	4 (3.31%)	2 (6.06%)	
≤5	117 (96.69%)	31(93.94%)	
CA125 (U/mL)			0.54
>24	20 (16.53%)	4 (12.12%)	
≤24	101 (83.47%)	29 (87.88%)	
CA199 (U/mL)			0.60
>25	35 (28.93%)	8(24.24%)	
≤25	86 (71.07%)	25 (75.76%)	
INR unit	1.04 ± 0.11	1.07±0.11	0.19
ALT (IU/L)	38.9 ± 41.03	43.39±50.39	0.60
AST (IU/L)	45.17 ± 44.27	43±49.3	0.28
**HCC lesion features**			
Number of lesions	124	33	
Size (cm)	4.25 ± 3.46	3.87 ±2.92	0.88

Note: Data are the mean ± standard deviation or median and interquartile range (IQR) unless otherwise indicated. HCC: hepatocellular carcinoma; BMI: body mass index; AFP: α-fetoprotein; CEA: carcinoembryonic antigen; CA125: carbohydrate antigen 125; CA199: Carbohydrate antigen 199; INR: international normalized ratio of prothrombin time; ALT: Alanine transaminase; AST: Aspartate transaminase.

* Indicates statistically significant *p* values.

Supplementary Table 3. Mechanical parameters *c* (stiffness) and *φ* (fluidity) of progenitor-type and non-progenitor-type HCC.

Parameters	Progenitor-type HCC (n = 51)	Non-progenitor-type HCC (n = 60)	*p* value
**Tumor**			
c (m/s)	2.10 ± 0.60	2.36 ± 0.61	0.02*
φ (rad)	1.09 ± 0.23	1.06 ± 0.21	0.72
**Liver**			
c (m/s)	1.93 ± 0.41	2.06 ± 0.43	0.11
φ (rad)	0.79 ± 0.12	0.77 ± 0.15	0.51

* Indicates statistically significant *p* values.

Supplementary Figure 1. Bland-Altman plots of reader agreement. The *c* (**a**) and *φ* (**b**) values of the tumor and *c* (**c**) and *φ* (**d**) values of the liver.

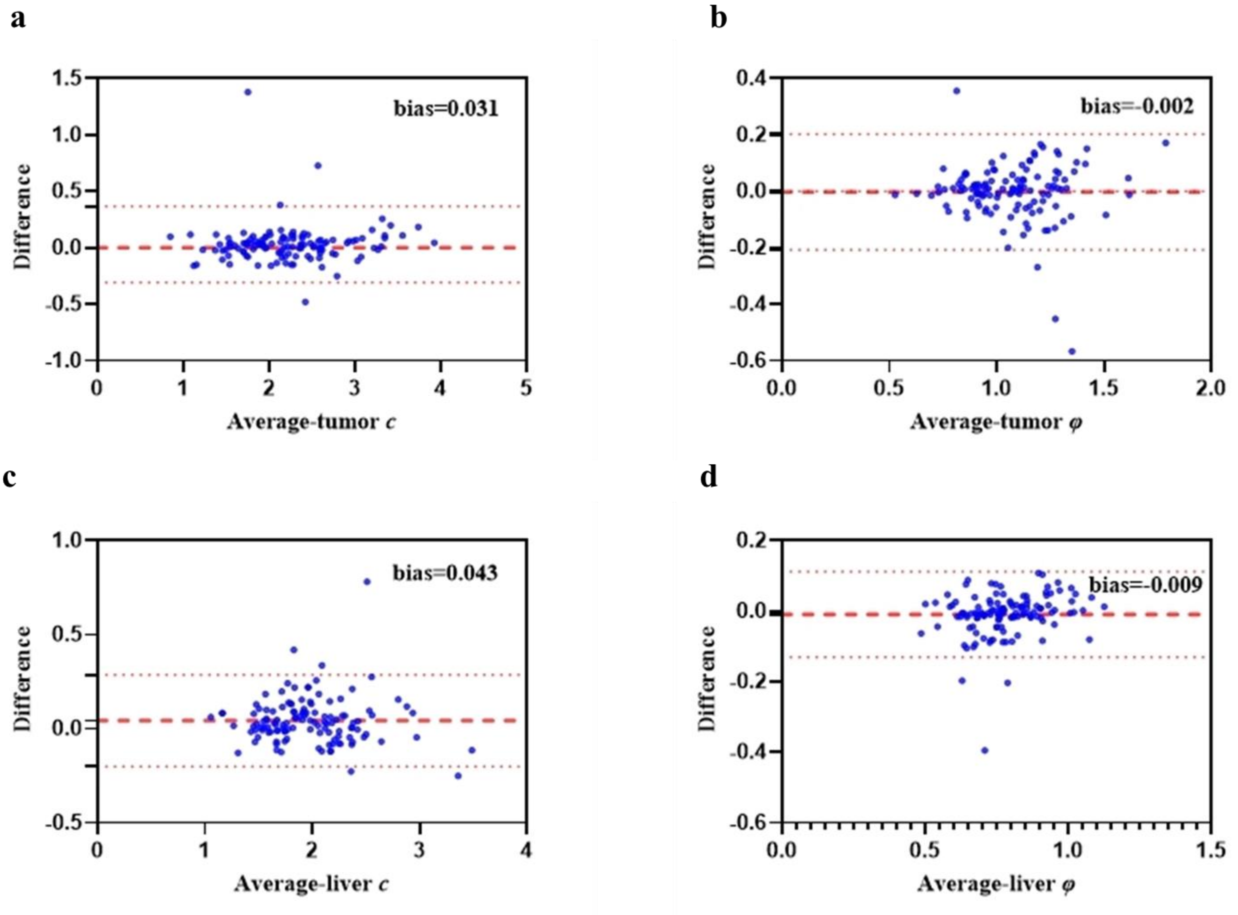

Supplement: Supplementary file 1 — Additional file 1. Supplementary Table 1. Multiparametric MRI protocol. Supplementary Table 2. Demographics and clinical characteristics of the participants in this study. Supplementary Table 3. Mechanical parameters c (stiffness) and φ (fluidity) of progenitor-type and non-progenitor-type HCC. Supplementary Figure 1. Bland-Altman plots of reader agreement. The c (a) and φ (b) values of the tumor and c (c) and φ (d) values of the liver. [file 13244_2023_1427_MOESM1_ESM.pdf]
